# SUMO-Specific Cysteine Protease 1 Promotes Epithelial Mesenchymal Transition of Prostate Cancer Cells via Regulating SMAD4 deSUMOylation

**DOI:** 10.3390/ijms18040808

**Published:** 2017-04-12

**Authors:** Xiaoyan Zhang, Hao Wang, Hua Wang, Fengjun Xiao, Prem Seth, Weidong Xu, Qinghua Jia, Chutse Wu, Yuefeng Yang, Lisheng Wang

**Affiliations:** 1 Department of Experimental Hematology, Beijing Institute of Radiation Medicine, Beijing 100850, China; zhangxy1120@aliyun.com (X.Z.); wang-home163@163.com (H.W.); wanghua@bmi.ac.cn (H.W.); xiaofjun@sina.com (F.X.); jjiaqh@hotmail.com (Q.J.); wuct@bmi.ac.cn (C.W.); 2 Gene Therapy Program, Department of Medicine, NorthShore Research Institute, Evanston, IL 60201, USA; PSeth@northshore.org (P.S.); wdxuii@gmail.com (W.X.)

**Keywords:** SENP1, deSUMOylation, EMT, SMAD4, E-cadherin, prostate cancer

## Abstract

In advanced prostate cancer, small ubiquitin-like modifier (SUMO)-specific cysteine protease 1 (SENP1) is up-regulated. However, the role of SENP1 in regulating deSUMOylation of TGF-β/SMADs signaling is unknown. In this study, we developed a lentiviral vector, PLKO.1-shSENP1, to silence SENP1 in prostate cancer cells with high metastatic characteristics (PC3M). Likewise, we also created an adenovirus vector, Ad5/F11p-SENP1 to over-express SENP1 in prostate cancer cells with low metastatic potential (LNCaP). We showed that silencing of SENP1 promoted cellular apoptosis, and inhibited proliferation and migration of PC3M cells. Moreover, SENP1 silencing increased the SMAD4 expression at protein level, up-regulated E-cadherin and down-regulated Vimentin expression, indicating the inhibition of epithelial mesenchymal transition (EMT). Furthermore, SMAD4 interference abolished SENP1-mediated up-regulation of E-cadherin, suggesting that SENP1 regulated E-cadherin expression via SMAD4. SENP1 over-expression in LNCaP cells reduced SMAD4 protein, and promoted EMT via decreasing E-cadherin and increasing Vimentin. Moreover, down-regulation of SMAD4 and E-cadherin were blocked, after transfection with two SUMOylation sites mutated SMAD4, suggesting that SENP1 might reduce SMAD4 levels to regulate E-cadherin expression via deSUMOylation of SMAD4. In conclusion, SENP1 deSUMOylated SMAD4 to promote EMT via up-regulating E-cadherin in prostate cancer cells. Therefore, SENP1 is a potential target for treatment of advanced prostate cancer.

## 1. Introduction

Small ubiquitin-like modifier (SUMO) is an ubiquitin-like protein, and SUMOylation regulates many cellular events, including nuclear signaling, transcription activities, and DNA repair [1,2]. SUMOylation is a dynamic process, and can be reversed by SUMO-specific cysteine proteases (SENPs). One such protease SENP1 has been widely investigated in many cancers including prostate cancer, breast cancer and colon cancer [3,4]. Prostate cancer is the most commonly diagnosed cancer in United States and is also on rapid rise in China [5]. It has been reported that SENP1 is up-regulated in prostate cancer patients, and promotes both androgen receptors (ARs)-dependent and ARs-independent cell proliferation [6,7]. Importantly, high levels of SENP1 have been linked to advanced pathological stages, higher Gleason grade, positive lymph node status, and prostate specific antigen (PSA) recurrence [8]. Some reports have shown that SENP1 stabilizes hypoxia inducible factor 1 (HIF-1α) to promote tumor growth and metastasis, and increases vascular endothelial growth factor (VEGF) expression to increase angiogenesis in the tumors [9,10].

Transforming growth factor β (TGF-β), known as a pleiotropic cytokine in regulating various biological processes, plays dual roles in the cancer development and progression. Under physiological condition, TGF-β exerts biological activities, such as the inhibition of cell proliferation, and induction of cell apoptosis, via TGFβ/SMADs signaling pathways. However, during cancer progression, TGF-β/SMADs signaling-mediated growth inhibition is generally blocked, due to the loss and inactivition of the mother against decapentaplegic homolog (SMAD) molecules [11]. Among the SMADs, SMAD4 is an important tumor suppressor, which has also been recognized as a potential molecular maker for diagnosis of prostate cancer [12,13]. It has been reported that SUMOylation of SMAD4 increases protein expression and stability of SMAD4, which can enhance the transcriptional activities of SMAD4 target genes [14,15]. However, the role of SENP1 in regulating deSUMOylation of SMAD4 in prostate cancer is largely unknown.

In this study, we silenced SENP1 in PC3M cells, a prostate cancer cell line with high metastatic potential, and over-expressed SENP1 in LNCaP cells, prostate cancer cells with low metastatic phenotype. Using these transduced cells, we examined the biological characteristics, SMAD4 protein, and epithelial mesenchymal transitions (EMT) markers, including E-cadherin and Vimentin. Then, the role of SENP1-mediated SMAD4 deSUMOylation in regulating E-cadherin expression was analyzed by SMAD4 silencing, as well as by introducing SMAD4 mutations in the SUMOylation sites.

## 2. Results

### 2.1. SENP1 Silencing Induces Apoptosis, Inhibits Cell Growth and Migration in PC3M, an Androgen-Independent Prostate Cancer Cell Line

**Figure 1:**
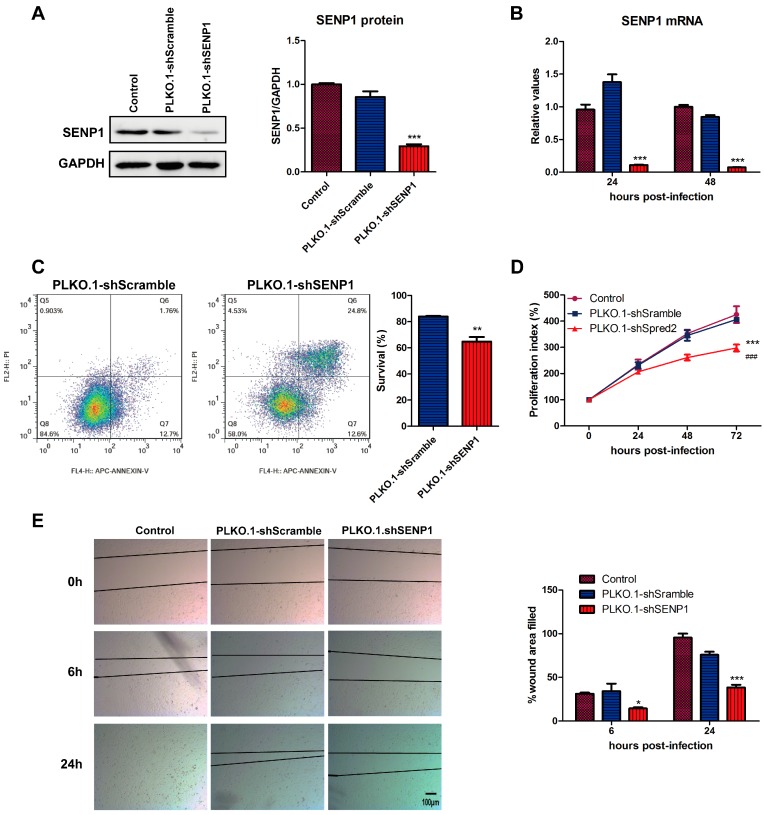
Effect of small ubiquitin-like modifier (SUMO)-specific cysteine protease 1 (SENP1) on the biological characteristics of PC3M prostate cancer cells. (**A**,**B**) Lentiviral vector mediated silencing of SENP1 in PC3M cells. PC3M cells were infected with 20 MOI (multiplicity of infection) of PLKO.1-shSENP1 or PLKO.1-shScramble. 48 h after infection, the SENP1 protein expression was detected by Western-blotting (**A**); At 24 and 48 h after infection, the total RNA was isolated and the mRNA expression of SENP1 was also analyzed by real-time reverse transcript polymerase chain reaction (RT-PCR) (**B**); (**C**) PLKO.1-shSENP1 induces apoptosis in PC3M cells. PC3M cells were infected with lentiviral vectors using 20 MOI. Forty-eight hour later, cells were collected, labeled with Annexin-V-APC and cellular apoptosis was analyzed by flow cytometry; (**D**) Proliferation of PC3M cells transduced with lentiviral vectors. PC3M cells were labeled with dye670, and then infected with lentiviral vectors. At indicated time points after infection, cells were collected and analyzed by flow cytometry. The proliferation index was calculated, using uninfected PC3M cells as control; (**E**) PLKO.1-shSENP1 inhibits the migration of PC3M cells. Confluent PC3M cells were scratched to generate wounds, at 24 h following infection with lentiviral vectors. 6 and 24 h later, the percentages of wound area filled were determined and analyzed. All the data were obtained from at least three independent experiments, and are shown as mean ± s.e.m. * *p* < 0.05, ** *p* < 0.01, *** *p* < 0.001, vs. PLKO.1-shScramble group; ^###^ *p* < 0.001 vs. control group.

To study the effects of SENP1 on the biological effects of prostate cancer cells, we constructed a lentiviral vector expressing short hairpin RNA targeting SENP1, PLKO.1-shSENP1, and a control vector, PLKO.1-shScramble. Transduction of PC3M cells with PLKO.1-shSENP1 down-regulated the SENP1 expression, both at protein (Figure 1A) and mRNA level (Figure 1B). Then, the biological characteristics were analyzed in PC3M cells infected with lentiviral vectors. We found that PLKO.1-shSENP1-mediated SENP1 silencing induced cellular apoptosis (Figure 1C), inhibited cell proliferation (Figure 1D) and reduced cell migration (Figure 1E). These results suggest that SENP1 interference might be a potential therapeutic approach to inhibit tumor growth and prevent tumor metastasis.

### 2.2. SENP1 Interference Enhances TGF-β/Smads Signaling and Inhibits EMT in PC3M Cells

**Figure 2:**
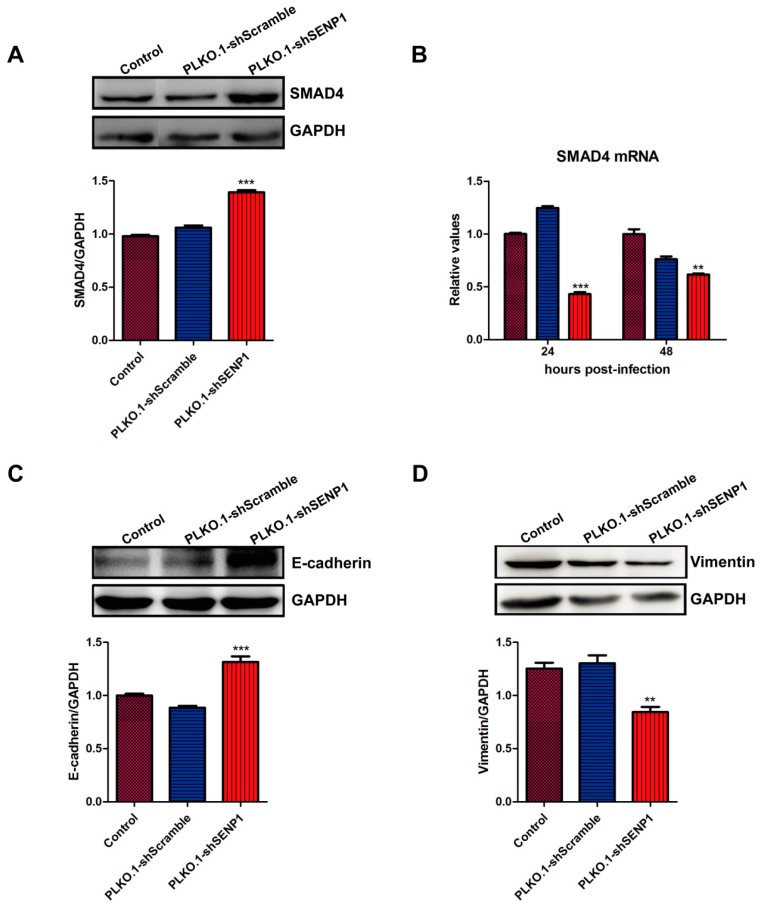
SENP1 interference enhances transforming growth factor (TGF-β)/SMADs signals, and inhibits epithelial mesenchymal transition (EMT) in PC3M cells. (**A**) PLKO.1-shSENP1 increases SMAD4 protein expression. PC3M cells were infected with 20 MOI PLKO.1-shSENP1 or PLKO.1-shScramble. 48 h later, cells were collected and SMAD4 protein was detected by Western-blotting; (**B**) SENP1 silencing decreased SMAD4 mRNA expression. At 24 and 48 h post-infection, cells were collected, and SMAD4 mRNA expression was detected by real-time RT-PCR; (**C**,**D**) SENP1 interference up-regulates E-cadherin protein, and reduces vimentin protein in PC3M cells. At 48h after infection with lentiviral vectors, protein expression of E-cadherin (**C**) and vimentin (**D**) was analyzed by Western-blotting as described above. All the data were obtained from at least three independent experiments, and are shown as mean ± s.e.m. ** *p* < 0.01, *** *p* < 0.001, vs. PLKO.1-shScramble group.

SMAD4 can be SUMOylated to regulate expression of TGF-β target genes. To test if SENP1 could deSUMOylate SMAD4 in prostate cancer cells, we analyzed SMAD4 expression in PC3M cells after infection with PLKO.1-shSENP1 or PLKO.1-shScramble. Interestingly, SENP1 silencing increased the expression of SMAD4 at the protein level (Figure 2A), but not at the mRNA level (Figure 2B), which suggested that SENP1 regulates the protein expression of SMAD4 at post-translational level. Furthermore, SENP1 interference increased E-cadherin protein, and reduced vimentin protein expression, which indicated the inhibition of EMT (Figure 2C,D). This is consistent with previous reports that TGF-β could promote the EMT in various tumor cells.

### 2.3. SENP1 Over-Expression Impairs TGF-β/Smads Signaling and Promotes EMT of Androgen-Dependent Prostate Cancer Cells, LNCaP

**Figure 3:**
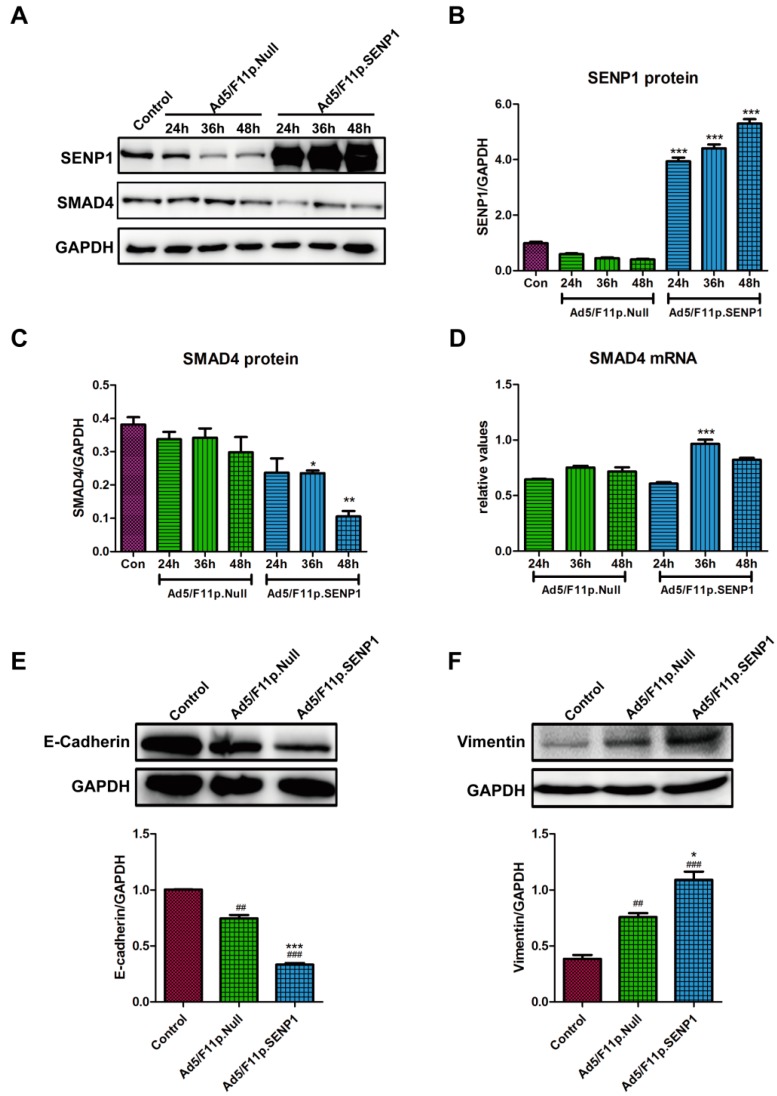
SENP1 over-expression decreases TGF-β/SMADs signals and promotes EMT of LNCaP cells. (**A**–**C**) SENP1 over-expression inhibits SMAD4 protein expression in LNCaP cells. LNCaP cells were infected with 10 MOI Ad5/F11p.SENP1 or Ad5/F11p.Null. At 24 h, 36 h and 48 h after infection, cells were collected and protein expression of SENP1 and SMAD4 was detected by Western-blotting (**A**), and the corresponding semi-quantitative results were shown in B and C respectively; (**D**) SENP1 increases SMAD4 mRNA expression in LNCaP cells. At 24 h, 36 h and 48 h after infection with adenoviruses, cells were collected, and SMAD4 mRNA expression was evaluated by real-time RT-PCR, and normalized by its expression in normal cultured LNCaP cells; (**E**,**F**) SENP1 reduces E-cadherin expression and promotes vimentin expression in LNCaP cells. 48 h after transduction with Ad5/F11p.SENP1 or Ad5/F11p.Null, the protein expression of E-cadherin (**E**) and vimentin (**F**) was detected by Western-blotting, and the semi-quantitative data are shown. All the data were obtained from at least three independent experiments, and are shown as mean ± s.e.m. * *p* < 0.05, ** *p* < 0.01, *** *p* < 0.001, vs. Ad5/F11p.Null group; ## *p* < 0.01, ### *p* < 0.001, vs. Ad5/F11p.Null group at the same time point.

To further investigate the effects of SENP1 on TGF-β/SMADs signals and EMT markers, a chemic fiber modified replication deficiency adenovirus, Ad5/F11p.SENP1, and control adenovirus, Ad5/F11p.Null were constructed. In low endogenous SENP1 expressing prostate cancer cells, LNCaP, Ad5/F11p.SENP1 infection produced SENP1 protein efficiently (Figure 3A,B). Moreover, SENP1 over-expression reduced SMAD4 protein expression at 48 h after infection (Figure 3A,C). However, the mRNA expression of SMAD4 was up-regulated at 36 h and 48 h post-infection (Figure 3D), which again suggested that SENP1 regulated the protein expression at post-translation level, in consistent with the results in PC3M cells. Moreover, SENP1 down-regulated E-cadherin protein and increased vimentin protein in LNCaP cells, at 48 h after Ad5/F11p-SENP1 transduction, indicating that SENP1 promoted the EMT of LNCaP cells (Figure 3E,F). Taken together, these studies suggest that in low-expressing SENP1 LNCaP cells, SENP1 over-expression down-regulated SMAD4 protein expression and promoted EMT of tumor cells.

### 2.4. SENP1 Regulates deSUMOylation of SMAD4 and Promotes EMT of Tumor Cells

**Figure 4:**
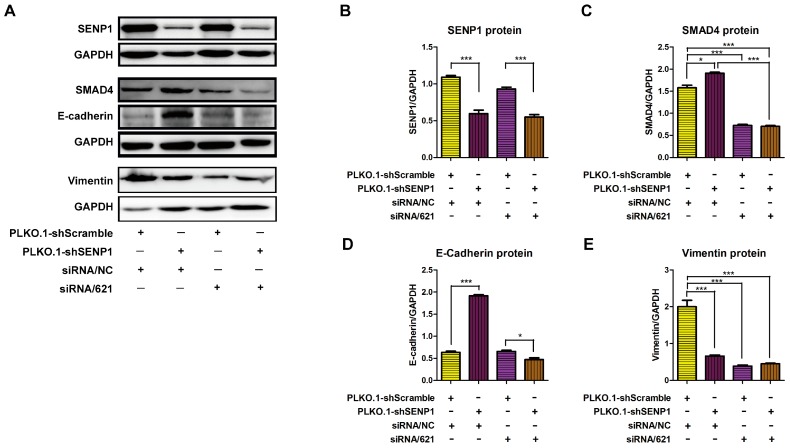

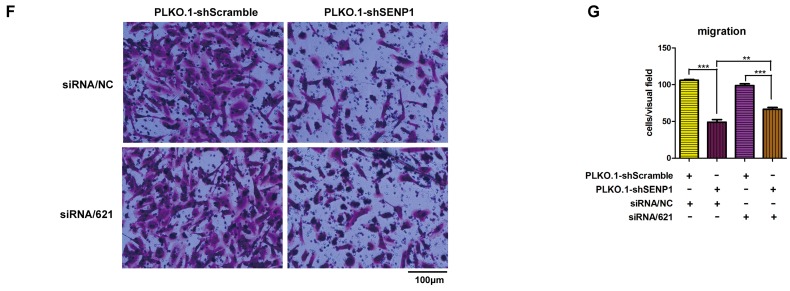
SMAD4 interference abolishes PLKO.1-shSENP1 induced E-cadherin expression in PC3M cells. PC3M cells were infected with 20 MOI PLKO.1-shScramble or PLKO.1-shSENP1. 24 h later, cells were transfected with siRNA targeting SMAD4 (siRNA/621) or control (siRNA/NC). After 48 h incubation, cells were collected and the protein expression of SENP1, SMAD4, E-cadherin and Vimentin was detected by Western-blotting (**A**). The semi-quantitative analysis of SENP1, SMAD4, E-cadherin and Vimentin was conducted, and are presented in (**B**–**E**), respectively. Moreover, the ability of migration was analyzed by transwell at 24 h after transfection with siRNAs. The representative images were shown in (**F**), and the statistical results were shown in (**G**). * *p* < 0.05, ** *p* < 0.01, *** *p* < 0.001, vs. corresponding group.

Our studies described above have shown that SMAD4 is up-regulated by SENP1 silencing in PC3M cells, while it is down-regulated in SENP1 over-expressing LNCaP cells. Next, we investigated if SENP1 regulates the EMT of tumor cells via controlling SMAD4 expression. Specific short interfering RNAs targeting SMAD4 were transfected into PC3M cells for analyzing interference efficiency. Among these, siRNA621 was the most promising one, and was selected for SMAD4 knockdown. siRNA621 was transfected into PC3M cells or SENP1-silenced PC3M cells (Figure 4A,B). We found that siRNA621 transduction reduced the SMAD4 protein expression in PC3M cells. Moreover, siRNA621 prevented SENP1 interference-mediated up-regulation of SMAD4 protein (Figure 4A,C). Importantly, at 48 h after transduction, siRNA621 abolished SENP1 silence induced E-cadherin expression (Figure 4A,D), suggesting that SENP1 silencing could elevate E-cadherin protein levels via increasing SMAD4 protein expression. Interestingly, both SENP1 interference and SMAD4 silencing reduced Vimentin expression in PC3M cells (Figure 4A,E). These results indicated that SENP1 silencing down-regulated Vimentin expression by other mechanisms, but not via up-regulating SMAD4 protein. Importantly, our data also showed that SMAD4 silencing could partly recover the ability of migration in SENP1 silenced PC3M cells (Figure 4F,G). Therefore, SMAD4 plays pivotal roles in SENP1-mediated regulation of E-cadherin expression, EMT and migration in PC3M cells.

**Figure 5:**
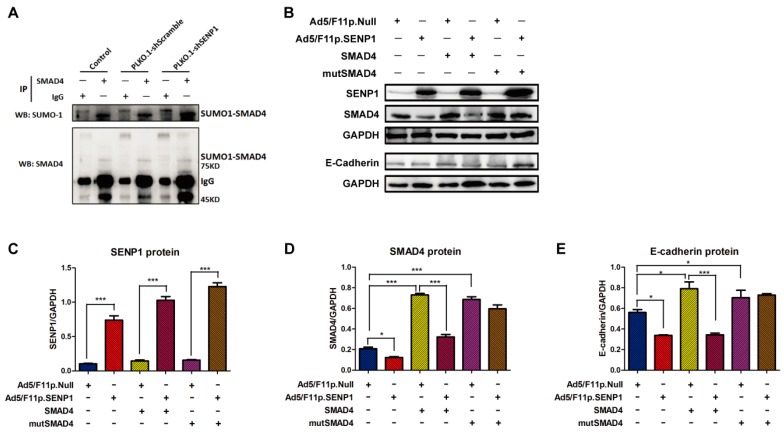
Mutations in SMAD4 at the SUMOylation sites of prevent SENP1-mediated degradation of SMAD4 and the down-regulation of E-cadherin. PC3M cells were transfected with lentiviral vectors, PLKO.1-shScramble and PLKO.1-shSENP1. 48 h later, cells were collected for immunoprecipitation analysis. The representative images were shown in (**A**). LNCaP cells were transfected with pcDNA3.0-SMAD4, pcDNA3.0-mutSMAD4 or pcDNA3.0 (control). 48 h later, cells were infected with 10 MOI Ad5/F11p.SENP1 or Ad5/F11p.Null. Cells were incubated for another 48 h, and then, the SENP1, SMAD4 and E-cadherin expression was detected by Western-blotting. The representative images are shown in (**B**); the semi-quantitative results of SENP1, SMAD4, and E-cadherin were analyzed and are presented in (**C**–**E**) respectively. All the data were obtained from at least three independent experiments, and are shown as mean ± s.e.m. * *p* < 0.05, *** *p* < 0.001, vs. corresponding group.

As described above, SENP1 could regulate SMAD4 protein expression at the post-translation level. Therefore, we investigated if SENP1 deSUMOylated SMAD4 in prostate cancer cells. In PC3M cells, we showed that PLKO.1-shSENP1 transfection could enhance the SUMOylation of SMAD4 via improving SUMO-1-SMAD4 complex (Figure 5A). To further confirm SENP1-mediated deSUMOylation of SMAD4, we constructed a plasmid vector pcDNA3.0-mutSMAD4 expressing SMAD4 with two mutation at SUMOylation sites, K113R and K159R; and using pcDNA3.0-SMAD4 as a control vector expressing wild type SMAD4. Both of these vectors were shown to produce SMAD4 protein in LNCaP cells (Figure 5A). Next, Ad5/F11p.SENP1 and Ad5/F11p.Null were transduced in SMAD4 over-expressing LNCaP cells (Figure 5B,C). We found that SENP1 could down-regulate SMAD4 protein level, both in control and wild SMAD4 over-expressing LNCaP cells. However, in mutated SMAD4 over-expressing LNCaP cells, SENP1 had no effect on SMAD4 expression, indicating that SENP1 could first deSUMOylate SMAD4 protein that leads to protein degradation (Figure 5B,D). Furthermore, SENP1 reduced E-cadherin expression both in the control and wild SMAD4 over-expressing LNCaP cells, but not in mutSMAD4 expressing LNCaP cells (Figure 5B,E). These results suggested that SENP1 could deSUMOylate and degrade SMAD4 protein to inhibit E-cadherin expression. Therefore, we propose that SENP1 up-regulation in prostate cancer cells deSUMOylates SMAD4, which in turn regulates E-cadherin, induces EMT of tumor cells and promotes tumor metastasis.

## 3. Discussion

In advanced stage of prostate cancer, a majority of patients develop distant metastasis, such as bone metastasis. Patients with metastases are insensitive to conventional treatments, including androgen-deprivation, chemotherapy and radiotherapy [16,17]. For bone metastatic patients, bisphosphonates and denosumab, a human monoclonal antibody against receptor activator of nuclear factor κ-B ligand (RANKL) could inhibit bone resorption and improve bone density to relieve pain and tumor-induced hypercalcemia [18,19]. However, the effective approaches to improve patients’ overall survival are still lacking. Therefore, it is urgent to discover novel and more promising therapeutic targets for advanced and metastatic prostate cancer.

TGF-β is considered to be a potent growth inhibitor under physiological conditions, and inhibits tumor growth at the early stage of cancers. However, during the advanced stages, TGF-β is aberrantly activated and cross-talks with several cell signaling pathways, both canonical and non-canonical, to facilitate tumor growth and metastases [20]. For example, TGF-β can increase VEGF expression via Src/Fak/Akt signaling to promote angiogenesis, and activate HIFs through PI3K/Akt/mTOR signaling to regulate metabolism and growth of tumor cells. Therefore, several TGF-β inhibitors have been developed, and show effective anti-tumor responses in animal models [21,22]. Our group has also developed oncolytic adenoviruses expressing soluble TGF-β receptor II fusion human IgG Fc fragment (sTGFβRIIFc), which can block TGF-β signaling and inhibit tumor bone metastasis [23,24].

In addition to crosstalk with several cell signaling pathways, which promotes tumor growth and metastasis, the blockade of TGF-β/SMADs-mediated growth inhibition and transcriptional activities also plays pivotal roles to tumor progression [11,20]. SMAD4, which lies downstream of SMAD2/3, is known to be an important tumor suppressor. It has been reported that down-regulation or inactivation of SMAD4 could promote the development and progression of prostate cancer, and SMAD4 has been emerged as a potential biomarker for diagnosis [12,13]. The SMAD4 levels are not only determined by transcription activities, but also influenced by post-translation modification, such as ubiquitinoylation, acetylization and SUMOylation [25,26]. Several reports have shown that SUMOylation could improve the stability and enhance the expression levels of SMAD4, which not only increases TGF-β-mediated transcription, but also negatively regulates ARs in prostate cancer [14,15]. However, SENP1, which could reverse the SUMOylation of protein, is significantly up-regulated in prostate cancers. In this study, we have shown that SENP1 silencing up-regulated SMAD4 in PC3M cells at protein level, but not at mRNA level, suggesting that SENP1 regulates SMAD4 expression at post-translation level. Moreover, in LNCaP cells, over-expression of SMAD4 protein with two mutations at SUMOylation sites could not be down-regulated by SENP1 over-expression. Therefore, we believe that SENP1 down-regulates SMAD4 protein through deSUMOylation.

EMT is an essential event during tumor metastasis to distant sites, and is associated with tumor cells to lose epithelial makers, such as E-cadherin, while acquiring mesenchymal makers, such as vimentin and N-cadherin. During advanced stages of cancers, TGF-β can induce EMT in tumor cells via both SMADs-dependent and -independent activation of EMT related transcription factors [27,28]. However, whether SENP1 participates in TGF-β induced EMT in prostate cancers has not been previously reported. In this study, we have shown that SENP1 silencing enhanced E-cadherin expression, while it inhibited vimentin expression in PC3M cells, indicating its role in the inhibition of EMT. We have also shown that the interference of SMAD4 could abolish this inhibitory effect of EMT. Taken together, these studies suggest that SENP1 silencing could inhibit the EMT of prostate cancer cells via up-regulating SMAD4 expression.

## 4. Materials and Methods

### 4.1. Cell Lines

Human androgen-independent prostate cancer cell, PC3M, was kindly provided by the Institute of Urology, Peking University (Beijing, China). Human androgen-dependent prostate cancer cell LNCaP was obtained from the Institute of Basic Medicine, Chinese Academy of Medicine Science (Beijing, China). Human embryonic kidney cell line HEK293 and 293T were purchased from American Type Culture Collection (ATCC, Manassas, VA, USA). All the cells were maintained in Dulbecco’s Minimal Essential Medium (DMEM, Gibco, Grand Island, NY, USA) supplemented with 10% fetal calf serum (FCS, Logan, UT, USA).

### 4.2. Vectors

Lentiviral short hairpin RNA (shRNA) vector targeting SENP1 (PLKO.1-shSENP1) was constructed according to the protocol of PLKO.1-puro vector (Addgene, Cambridge, MA, USA). Briefly, the forward oligo, 5′TCGAGCGCCAGATTGAAGAACTCGAGTTCTGTTCTTCAATCTGGCGCTTTTTG3′ and reverse oligo, 5′GATCCAAAAAGCGCCAGATTGAAGAACAGAACTCGAGTTCTGTTCTTCAATCTGGCGC3′ were annealed and inserted into the PLKO.1-puro vector. Control vector PLKO.1-shScramble was also purchased from addgene. Lentiviruses were produced in 293T cells after co-transfection of PLKO.1-shSENP1 or PLKO.1-shScramble, packing plasmid psPAX2 and envelope plasmid pMD2.G using the phosphate co-precipitation kit (Promega, Madison, WI, USA). Viruses were purified and concentrated by PEG, followed by determination of viral titers on HT1080 cells.

A chemic fiber-modified and replication-deficient adenovirus-expressing SENP1, Ad5/F11p.SENP1, and control vector, Ad5/F11p.Null, were constructed as previously described [29]. Wild type SMAD4 gene and mutated SMAD4 gene with two mutations (K113R and K159R) was synthesized (AuGCT, Beijing, China), and inserted into the multiple cloning sites to generate pcDNA3.0-SMAD4 and pcDNA3.0-mutSMAD4, respectively. siRNA621, targeting human SMAD4, and the corresponding control, siRNANC were purchased from GenePharma (Shanghai, China).

### 4.3. Biological Analysis of PC3M Cells after Infection with Lentiviruses

#### 4.3.1. Apoptosis Analysis

Exponentially growing PC3M cells were seeded into 6-well plate at a density of 2.0 × 10^5^ cells/well. 24 h later, cells were infected with PLKO.1-shSENP1 or PLKO.1-shScramble at 20 multiplicity of infection (MOI). After 24 h incubation, 1 mL of fresh complete culture media was added. 24 h later, cells were collected, labeled with APC conjugated Annexin-V and PI (Sungene Biotech Co., Ltd., Tianjin, China), and analyzed by flow cytometry on FACSCalibur (BD Bioscience, San Jose, CA, USA).

#### 4.3.2. Proliferation Assay

Exponentially growing PC3M cells were collected and labeled with Dye eFluor^®^ 670 (eBioscience, San Diego, CA, USA) according to the manufacturers’ instructions. Labeled cells were plated into 6-well plates at a density of 2 × 10^5^ cells per well. Next day, cells were infected with PLKO.1-shSENP1 and PLKO.1-shScramble at 20 MOI. At 0, 24, 48 and 72 h after infection, cells were collected and the fluorescence intensity of Dye 670 was measured by flow cytometry, and the proliferation index was calculated.

#### 4.3.3. Migration Assay

Exponentially growing PC3M cells were seeded into 6-well plate at a density of 2 × 10^5^ cells per well. Next day, cells were infected with 20 MOI of PLKO.1-shSENP1 and PLKO.1-shScramble, and the incubation continued for 24 h. Then, confluent cells were scratched to generate wounds, as described previously [30]. Cells were incubated for 0, 6, 24 h. Live cell images were taken, the distances between the two margins of the wounds were measured, and percentages of wound area filled were determined and analyzed as described previously [30].

For transwell assay, PC3M cells were infected with lentiviral vectors. 24 h later, cells were transfected with siRNAs. After another 24 h incubation, the migration ability of treated PC3M cells was examined by transwell. Briefly, the transwell insert was added to the well by merging the bottom of the insert into the medium in the lower compartment. Then, the cells were seeded into the transwell insert. After 20 h incubation, migrated cells were fixed, stained and counted.

### 4.4. Western-Blotting and Immunoprecipitation

48 h post-infection with PLKO.1-shSENP1 or PLKO.1-shScramble, the protein expressions of SENP1, SMAD4, E-cadherin and Vimentin in PC3M cells were detected by Western-blotting. Various antibodies used were rabbit anti-human SENP1 monoclonal antibody (Abcam, Cambridge, MA, USA), rabbit anti-human SMAD4 monoclonal antibody (Cell Signaling Technology, CST, Danvers, MA, USA), rabbit anti-human E-cadherin monoclonal antibody (Abcam), and rabbit anti-human Vimentin monoclonal antibody (Abcam). The relative expression levels were normalized by the glyceraldehyde-3-phosphate dehydrogenase (GAPDH). In LNCaP cells, the SENP1, SMAD4, E-cadherin and Vimentin expression were also detected by Western-blotting at 24, 36 and 48 h following infection with Ad5/F11p.SENP1 and Ad5/F11p.Null adenoviruses.

To analyze the role of SMAD4 in SENP1-mediated up-regulation of E-cadherin, PC3M cells were transduced with PLKO.1-shSENP1 and PLKO.1-shScramble. 24 h later, cells were transfected with siRNAs (siRNA621 or siRNANC), and the incubations continued for 48 h. Then, protein expression of SENP1, SMAD4, E-cadherin and Vimentin was analyzed by Western-blotting.

To investigate if SENP1 deSUMOylates SMAD4 to up-regulate E-cadherin, mutated SMAD4 expressing vector pcDNA3.0-mutSMAD4, wild type SMAD4 encoding vector pcDNA3.0-SMAD4 or control vector pcDNA3.0 were transfected into LNCaP cells. 24 h later, cells were infected with adenoviruses, Ad5/F11p.SENP1 or Ad5/F11p.Null. After 48 h incubation, the protein expression of SENP1, SMAD4 and E-cadherin were detected by Western-blotting.

At 48h after infection with PLKO.1-shScramble and PLKO.1-shSENP1, PC3M cells were used to conduct immunoprecipitation analsysis, using the primary antibodies anti-SMAD4 (CST), anti-SUMO-1 (CST) and anti-IgG, according to the manufacturer’s instructions.

### 4.5. Real-Time Reverse Transcript Polymerase Chain Reaction (RT-PCR)

PC3M cells infected with lentiviruses were collected at 24 and 48 h after infection. LNCaP cells were obtained at 24, 36 and 48 h post-infection with adenoviruses. Then, total RNA was extracted, and cDNA was synthesized by using RevertAid First Strand cDNA Synthesis Kit (Thermo Scientific, Wilmington, DE, USA). The mRNA expressions of SENP1 and SMAD4 were quantified by using SYBR^®^ Premix Ex Taq™ (Tli RNaseH Plus) (Takara, Shiga, Japan) on 7500 Fast Real-Time PCR System (Applied Biosystems/Life Technologies, Foster City, CA, USA). The relative expression levels were calculated by 2^−Δ*C*t^, using β-actin as the control. The primers for SENP1 and SMAD4 were as following, SENP1: forward, 5′ATCAGGCAGTGAAACGTTGGAC3′ and reverse, 5′GCAGGCTTCATTGTTTATCCCA3′; SMAD4: forward, 5′GGACTGCACCATACACCT3′ and reverse, 5′AATGGGCTGGAATGCAA3′; and β-actin: forward, 5′CATCCTCACCCTGAAGTACCC3′ and reverse, 5′AGCCTGGATAGCAACGTACATG3′.

### 4.6. Statistical Analysis

Data are presented as mean ± s.e.m. and statistically analyzed by using GraphPad Prism software version 5 (GraphPad software, San Diego, CA, USA). One-way ANOVA followed by Bonferroni post hoc tests were performed to analyze multiple groups. Difference were considered significant at two sided *p* < 0.05.

## 5. Conclusions

Up-regulation of SENP1 promotes deSUMOylation of SMAD4 in prostate cancer cells, which reduces the SMAD4 levels and impairs TGF-β/SMADs-mediated transcription activities. SENP1 silencing leads to the increased expression of E-cadherin, and inhibition of EMT of tumor cells. We believe SENP1 silencing leads to the increased SMAD4 levels due to improved stability of SMAD4 in the tumor cells. Moreover, silencing of SENP1 promoted apoptosis, and inhibited the proliferation and migration of tumor cells. Therefore, SENP1 should be considered as a potential target for the treatment of advanced and metastatic prostate cancer.
